# Addendum: Bassan, J.C.; et al. Immobilization of Trypsin in Lignocellulosic Waste Material to Produce Peptides with Bioactive Potential from Whey Protein. *Materials* 2016, *9*(5), 357

**DOI:** 10.3390/ma9080705

**Published:** 2016-08-19

**Authors:** Juliana Cristina Bassan, Thaís Milena de Souza Bezerra, Guilherme Peixoto, Clariana Zanutto Paulino da Cruz, Julián Paul Martínez Galán, Aline Buda dos Santos Vaz, Saulo Santesso Garrido, Marco Filice, Rubens Monti

**Affiliations:** 1 Faculdade de Ciências Farmacêuticas, UNESP—Univ. Estadual Paulista, 14800-903, Departamento de Alimentos e Nutrição, Araraquara-SP, Brazil; julianacbassan@gmail.com (J.C.B.); jpaulmg1@yahoo.es (J.P.M.G.); 2 Instituto de Química, UNESP—Univ. Estadual Paulista, 14800-060, Departamento de Bioquímica e Tecnologia Química, Araraquara-SP, Brazil; milenatsb@gmail.com (T.M.d.S.B.); clarianadacruz@gmail.com (C.Z.P.d.C.); alinebuda@zootecnista.com.br (A.B.d.S.V.); saulosantesso@iq.unesp.br (S.S.G.); 3 Faculdade de Ciências Farmacêuticas, UNESP—Univ. Estadual Paulista, 14800-903, Departamento de Bioprocessos e Biotecnologia, Araraquara-SP, Brazil; 4 Fundación Centro Nacional de Investigaciones Cardiovasculares Carlos III, Melchor Fernández Almagro, Madrid 28029, Spain; marco.filice@cnic.es

The authors would like to add the following sentence to the “Acknowledgments” section of their article [1]:

“The authors thank Programa de Apoio ao Desenvolvimento Científico da Faculdade de Ciências Farmacêuticas da UNESP-PADC for their financial support”.
